# Improving Health and Diabetes Self-Management in Immigrants with Type 2 Diabetes Through a Co-Created Diabetes Self-Management Education and Support Intervention

**DOI:** 10.1007/s10900-022-01151-y

**Published:** 2022-11-03

**Authors:** Nana Folmann Hempler, Charlotte Fagt, Kasper Olesen, Sabina Wagner, Lone Banke Rasmussen, Ditte Hjorth Laursen, Charlotte Glümer, Mette Nygaard, Ingrid Willaing

**Affiliations:** 1 Health Promotion Research, Copenhagen University Hospital—Steno Diabetes Centre Copenhagen, Borgmester Ib Juuls Vej 89, 2730 Herlev, Denmark; 2 Liva Healthcare, 1434 Copenhagen, Denmark; 3 Centre for Diabetes, Municipality of Copenhagen, 1620 Copenhagen, Denmark; 4 Department of Health Science and Technology, Aalborg University, 9220 Aalborg, Denmark; 5 Department of Public Health, University of Copenhagen, 1014 Copenhagen, Denmark

**Keywords:** Immigrants, Cultural sensitivity, Co-creation, Diabetes self-management, Type 2 diabetes

## Abstract

To examine the impact of a co-created culturally sensitive diabetes self-management education and support (DSMES) intervention on the physical and mental health of immigrants with type 2 diabetes (T2D). Pre- and post-test among people with T2D whose primary language was Urdu, Arabic or Turkish (*n* = 97). Participants were offered a six-week intervention based on a person-centred approach using research-based dialogue tools to facilitate learning and reflection, which was developed in co-creation with immigrants and healthcare professionals. Data were collected at baseline, post-intervention and after 6 months and analysed using paired *t*-tests, Wilcoxon signed-rank tests, chi-square tests and regression models when appropriate. Several clinical outcomes were improved post-intervention, including HbA1c (*P* < 0.001), body fat percentage (*P* = 0.002), self-rated general health (*P* = 0.05), well-being (*P* = 0.004) and several self-management behaviours, e.g., physical activity (*P* < 0.001). Most outcomes remained improved after 6 months, but the effect on HbA1c was no longer statistically significant. Some outcomes were improved only at 6 months, including waist circumference (*P* < 0.001) and diabetes-related emotional distress (*P* < 0.001). Fatigue did not change. Attendance at more programme sessions was associated with better outcomes. The DSMES intervention developed in a co-creation process was highly effective in improving the health of immigrants with T2D.

## Introduction

Diabetes self-management education and support (DSMES) is a cornerstone of effective type 2 diabetes (T2D) treatment because it facilitates knowledge and skills that are essential for implementing and sustaining self-care behaviours [1, 2]. Attendance at DSMES programmes is associated with increased quality of life, reduced morbidity and mortality, and improved glycaemic control in populations with type 2 diabetes [1, 2]. However, immigrants with type 2 diabetes attend DSMES less often and benefit from it less than do other populations [3–9]. This may be attributed to the fact that traditional DSMES interventions do not often take into account differences in cultural beliefs and language abilities [10, 11]. Lack of cultural sensitivity, in addition to communication difficulties, lack of referrals, unfamiliarity in navigating the healthcare system, and stigmatisation may be a barrier for immigrants when accessing DSMES [12].

Several factors indicate that culturally sensitive DSMES interventions are needed. European studies indicate that immigrant groups born in non-Western countries have a higher risk of T2D, compared to the majority population [13–15]. In addition, immigrant groups and their descendants are at increased risk of developing complications of diabetes [7, 16]. In Denmark, the incidence of T2D and risk of both any type of retinopathy and diabetic retinopathy requiring referral are higher among immigrants born in Asia, Sub-Saharan Africa and the Middle East, compared with native Danes [17, 18].

Few studies have investigated the potential effects of culturally sensitive DSMES specifically targeting immigrant populations in a European context [19]. No established definitions of cultural sensitivity or cultural appropriateness exist, and cultural sensitivity exemplified in studies may vary in dose, content and format. According to Resnicow et al. [20], cultural sensitivity is the incorporation of the ethnic/cultural characteristics, experiences, norms, values, behavioural patterns, beliefs and environmental and social forces of the target population into the design, delivery, and evaluation of interventions. It includes surface structures, which match intervention content to observable characteristics of the target population, e.g., language or food preferences and deep structures reflecting an understanding of cultural, social, historical, environmental, and psychological forces influencing a specific health behaviour in the target population, such as fasting practices and family involvement [20].

Active involvement of the target group through co-creation in development is a way to ensure that a DSMES intervention meets the needs, preferences and resources of the target group [21]. Co-creation processes can increase the cultural sensitivity of interventions, which may lead to improved health outcomes [20].

This study explores the impact of a co-created and culturally sensitive group-based DSMES intervention, CUlturally Sensitive TOols and Methods (CUSTOM), on clinical outcomes, self-reported physical and mental health and self-management behaviours. The target population consisted of Urdu, Arabic and Turkish-speaking immigrants with T2D and limited skills in speaking Danish.

## Methods

### Study Design

CUSTOM is a complex intervention consisting of interacting components in the design, complex behaviour changes in those delivering and receiving the intervention and many stakeholders [22]. It examines impact using a pre- and post-test design. Data were collected at baseline (T1), post-intervention (T2) and at 6 months follow up (T3).

### Recruitment and Inclusion Criteria

Participants were referred to diabetes rehabilitation by their general practitioners and were recruited at a diabetes centre in Copenhagen, Denmark. Recruitment took place in March 2019–June 2021. At their first consultation, individuals were asked to participate in the study if an interpreter was needed. Those interested in participating were contacted by a research nurse who screened them for study eligibility.

Inclusion criteria were age ≥ 18 years, clinical diagnosis of T2D, ability to give written consent, ability and willingness to attend and participate in a group-based education intervention and Arabic, Turkish or Urdu as first language. Potential participants were excluded if they had a physical illness that could substantially reduce life expectancy, serious mental illness, significant alcohol or substance misuse or a primary diagnosis of a learning disability or were pregnant, breastfeeding or planned to become pregnant during the study period. All participants were offered written and audiotaped information about the study in their preferred language (Danish, Arabic, Turkish or Urdu).

### The CUSTOM Intervention

The DSMES intervention consisted of 6 weekly 2.5-h group sessions. The purpose was to support health-promoting decisions and improve daily diabetes self-management, and the intervention covered diabetes knowledge and complications, mental health, diet, exercise, blood sugar measurement and fasting. Eleven dialogue tools with images, cases, and illustrations and a comprehensive guide were developed to facilitate dialogue, learning, and reflection and promote diabetes-specific knowledge in group sessions. The intervention was delivered by an interdisciplinary team consisting of a nurse, dietician, physiotherapist and translator. Educators were trained in the theoretical foundation of CUSTOM, including cultural competence [23], a flourishing approach [24] and active listening [25]. Cultural competence includes awareness of social contexts and prejudices, the ability to transfer information to different patient groups and being flexible and creative in new situations [23]. The flourishing approach focuses on paying attention to patients’ strengths, what is going well, and successful experiences and approaches [24]. Active listening involves mirroring, in which health care professionals repeat what participants say and discuss the message of the communication with the patient [25].

CUSTOM was developed in co-creation with members of the target group, researchers from Steno Diabetes Centre Copenhagen and healthcare professionals from the Centre for Diabetes. The methodology of design thinking was used, which is a participatory approach in three phases: inspiration, ideation, and implementation [21, 26]. The project first entailed a needs assessment based on reviewing the literature, fieldwork and workshops with the target group and health care professionals. Ideation and development of the intervention were then undertaken with the active involvement of the target group in developing a prototype and prioritising and refining methods, materials, research tools and educational format. The final design phase consisted of prototype testing and refinement before implementation in practice. In workshops, the target group and health care professionals were actively involved in ideation, designing, testing, and prioritising final materials, methods, content and educational format. They also provided feedback about data collection, such as study information sheets and questionnaires. Throughout the design process, co-creation with the target group maintained a focus on cultural sensitivity in terms of both surface structure and deep structure. The intervention content and format are described in detail elsewhere [27].

### Data Collection

At baseline visits, sociodemographic and background information were obtained from participants. Physical examinations and questionnaires were conducted by the research nurse 2 weeks or less before participants began the DSMES intervention, 2 weeks or less after the DSMES intervention ended and at approximately 6 months (± 21 days) after the intervention ended. The research nurse recorded participants’ questionnaire responses while they viewed the questions on printed versions. Questionnaires were translated into Arabic, Turkish and Urdu following international standards by submitting the questionnaire to forward- and back-translations and review by an academic committee [28].

### Measures

#### Clinical Measures

##### HbA1c and Lipid Profile

A fingerstick blood sample of one drop was used to measure triglycerides (TG), total cholesterol (TC), high-density lipoprotein cholesterol (HDL-C), low-density lipoprotein cholesterol (LDL-C) and glycated hemoglobin (HbA1c) measured on Alere Afinion™ HbA1c (Abbott, Chicago, IL). A strict protocol was followed for collecting blood samples and, to ensure accuracy, the HemoCue analyser was calibrated daily according to the manufacturer’s user instructions. An additional calibration was carried out monthly using a special test kit to verify measurement sensitivity and accuracy.

Bodyweight to the nearest 0.1 kg was measured with participants wearing lightweight indoor clothes and no shoes. Height without shoes to the nearest 0.1 cm was measured with a transportable wall-mounted stadiometer. Waist circumference to the nearest 0.5 cm was measured halfway between the lowest point of the costal margin and the highest point of the iliac crest at the end of expiration with participants in a standing position wearing light indoor clothing. Hip circumference to the nearest 0.1 cm was measured at the level of the greater femoral trochanter and measured at the end of expiration with participants standing. All anthropometrics were measured twice, and means were used in analyses. Body mass index (BMI) was calculated as weight/height^2^. Waist/hip circumference was calculated as waist circumference/hip circumference. Blood pressure was measured with participants sitting after at least 10 min of rest and without talking during measurement, which was repeated 3 times at two-minute intervals. The mean value was calculated and used in analyses. Body fat percentage was measured using the DC-360 body composition analyser (TANITA, Tokyo).

#### Questionnaire

##### Sociodemographic and Diabetes-Related Measures

Language, cohabitation status, educational level, employment status, years living in Denmark and comorbidity were assessed in the baseline questionnaire. Age, gender, diabetes duration, prescribed oral antihyperglycemic medication (types and dosages), antihypertensive medication (types and dosages), cholesterol-lowering medication (types and dosages), diabetes complications, and participation in intervention sessions were obtained from medical records.

##### Self-Reported Physical and Mental Health

Self-rated general health was measured using one item from the 12-item short-form health survey (SF-12) [29]. Well-being was measured using the WHO-5, which comprises five items measuring different aspects of well-being during the past 2 weeks [30]. Perceived diabetes-related emotional distress was measured using a short form of the Problem Areas in Diabetes (PAID-5) questionnaire [31]. Fatigue was measured with the Fatigue Assessment Scale (FAS), 10 items assessing symptoms of chronic fatigue [32]. When responses were missing on one or two items on WHO-5, PAID-5 or FAS, missing values were replaced by the mean value for the item among remaining participants. When three or more responses were missing, individuals were excluded from analyses including these scales.

##### Health Behaviours and Diabetes Self-Management

Smoking habits and alcohol consumption were measured using questions from the Danish National Health Profile [33, 34]. Diabetes self-management activities were measured using selected items from the Summary of Diabetes Self-Care Activities (SDSCA) scale [35].

### Statistical Analysis

Descriptive statistics were used to assess baseline population characteristics. Changes between baseline and the end of the intervention and between baseline and 6 months were assessed with paired *t*-tests for approximately normally distributed continuous data, Wilcoxon signed-rank tests for non-normally distributed data and chi-square tests for categorical data. Results were expressed as means and standard deviations (SD) for normally distributed continuous data, medians and inter-quartile range (IQR) for non-normally distributed continuous data and counts and percentages for categorical data. Participants were included in analyses if they had a baseline assessment and an assessment at the end of the intervention or at 6 months. The relationship between DSMES attendance and outcome measures was assessed with general linear models (GLM) for continuous measures and logistic regression model for categorical measures. Residuals were approximately normally distributed, and variance in GLM estimates was generally homogenous. Statistical analyses were performed using SAS Studios version 3.8. The level of significance was set at *P* < 0.05 for all tests.

### Implications of the COVID-19 Pandemic on the Study

The COVID-19 pandemic had some implications for the study. Initially, the intention was to include a control group with individuals in the target group attending other DSMES interventions in five municipalities in the Capital Region, Denmark. However, the pandemic substantially limited both recruitment to and execution of education interventions in the municipalities; as a result, data from the control group could not be included in the study.

## Results

### Sample Characteristics

A total of 120 individuals were invited to participate in the intervention, of whom 97 participated in the baseline assessment. Twelve were lost to follow up between baseline and the end of the intervention, while another 12 were lost to follow up after 6 months, resulting in a response rate of 75%.

Participants included more women than men, and mean age was 59 years. Most participants had no or limited education and were out of work (Table 1). One in five participants had been diagnosed with type 2 diabetes less than a year previously, while two in five had been diagnosed with diabetes more than 10 years previously. Most participants were prescribed antihyperglycemic medications, and the majority reported having both diabetes complications and comorbid conditions. Sixty-one (63%) participants completed five or six sessions of the intervention and 69 (71%) completed at least four sessions.

**Table 1:** Participant characteristics (n = 97)

Variable	N	%	Mean ± SD
Sex			
Female	75	73.3	
Male	22	22.7	
Age			59.0 ± 10.0
Language			
Arabic	53	54.6	
Urdu	34	35.1	
Turkish	10	10.3	
Education			
None	19	19.59	
≤ 7 years of primary school	25	25.77	
Completed primary school	32	32.99	
Completed secondary school (high school)	21	21.65	
Education higher than primary or secondary (high school)			
None or currently studying	71	73.2	
One or more shorter courses	11	11.34	
Vocational	5	5.15	
Tertiary education (middle, intermediate, higher)	10	10.31	
Employment status			
Employed	6	6.2	
Out of work	91	93.8	
Years living in Denmark			30.1 ± 11.5
Cohabitation status			
Living with others	79	81.4	
Living alone	18	18.6	
Baseline HbA1c			58.9 ± 17.52
Diabetes duration^a^			
< 1 year	19	20.0	
2–3 years	12	12.6	
4–5 years	10	10.5	
6–10 years	16	16.8	
> 10 years	38	40.0	
Diabetes complications/problems			
Yes	67	69.1	
No/do not know	30	30.9	
Comorbidity			
Yes	55	56.7	
No/do not know	42	43.3	
Prescription of diabetes medications			
Tablets			
Yes	89	91.8	
No/do not know	8	8.2	
Injections			
Yes	25	25.8	
No/do not know	72	74.2	
Prescription of antihypertensive medication			
Yes	57	58.8	
No/do not know	40	41.2	
Prescription of cholesterol-lowering medication			
Yes	68	70.1	
No/do not know	29	29.9	
Alcohol			
Never	90	92.8	
Occasionally	7	7.2	
Smoking			
Yes	12	12.4	
No/former	85	87.6	
DSMES sessions attended, n			
0	5	5.1	
1	5	5.1	
2	10	10.3	
3	8	8.3	
4	8	8.3	
5	21	21.7	
6	40	41.2	

^a^2 missing

### Clinical Outcomes, Self-Reported Physical and Mental Health and Self-Management

#### Changes from Baseline to Intervention End

Most clinical outcomes improved from baseline to the end of the intervention; HbA1c, weight, BMI, total cholesterol/HDL-C ratio, and body fat percentage improved significantly (Table 2). With regard to mental and physical health and self-management, significant improvements were seen in self-rated general health, well-being, and self-management activities of healthy diet, physical activity, and foot care. Nonsignificant improvements were observed for diabetes-related emotional distress, fatigue and checking blood sugar from baseline to end of intervention.

**Table 2:** Changes in outcome measures from baseline to end of intervention

		Mean (SD)		Mean change (SD)	*P*-value
	N	Baseline	End		
HbA1c (mmol/mol)	85	58.6 (17.2)	56.7 (15.4)	− 1.91 (4.32)	< 0.001
Weight (kg)	85	81.4 (16.0)	80.9 (15.8)	− 0.53 (1.42)	< 0.001
BMI (kg/m^2^)	85	32.1 (6.0)	31.9 (5.9)	− 0.22 (0.56)	< 0.001
Waist circumference (cm)	85	103 (12.2)	103 (11.8)	− 0.53 (3.26)	0.14
Hip circumference (cm)	84	107 (11.93)	107 (11.6)	− 0.27 (2.70)	0.35
Waist/hip circumference ratio	84	0.97 (0.09)	0.96 (0.09)	− 0.003 (0.03)	0.47
Systolic BP (mm Hg)	82	137 (16.20)	136 (16.80)	− 0.84 (11.0)	0.49
Diastolic BP (mm Hg)	82	82.1 (9.92)	81.8 (9.60)	− 0.30 (6.80)	0.70
Total cholesterol (mmol/L)	81	4.28 (1.1)	4.21 (1.0)	− 0.08 (0.66)	0.30
HDL-C (mmol/L)	79	1.22 (0.30)	1.23 (0.30)	0.01 (0.14)	0.61
LDL-C (mmol/L)	68	1.97 (0.87)	1.93 (0.80)	− 0.04 (0.55)	0.52
Total cholesterol/HDL-C ratio	79	3.61 (0.94)	3.52 (0.95)	− 0.10 (0.38)	0.03
Triglycerides (mmol/L)	81	2.56 (1.27)	2.55 (1.60)	− 0.01 (1.40)	0.94
Body fat percentage (%)	83	39.6 (7.82)	39.2 (7.62)	− 0.40 (1.17)	0.002
Self-reported general health	84	3.44 (0.86)	3.24 (0.83)	− 0.20 (0.92)	0.05
WHO-5 score	85	45.8 (22.5)	52.2 (23.1)	6.38 (19.7)	0.004
PAID-5 score	71	9.60 (5.55)	8.70 (6.05)	− 0.91 (4.62)	0.10
Fatigue Assessment Scale score	69	33.0 (9.61)	32.0 (9.62)	− 1.01 (6.25)	0.18
SDSCA, median (IQR)					
Healthy diet	85	4 (3–7)	7 (3–7)		0.002
Physical activity	85	4.5 (1–7)	7 (3–7)		< 0.001
Foot care	85	7 (0–7)	7 (3–7)		0.002
Checking blood sugar	85	1 (0–7)	2 (0–5)		0.96
Taking diabetes medication	85	7 (7–7)	7 (7–7)		1.00

*HbA1c* glycated haemoglobin, *BMI* body mass index, *BP* blood pressure, *TC* total cholesterol, *HDL-C* high-density lipoprotein cholesterol, *LDL-C* low-density lipoprotein cholesterol, *BFP* Body Fat Percentage, *WHO-5* World Health Organization-5 Well-Being Index, *PAID* Problem Areas in Diabetes, *SDSCA* Summary of Diabetes Self-Care Activities

#### Changes from Baseline to Follow-Up

After 6 months, weight, BMI, body fat percentage, waist circumference, hip circumference, and HDL-cholesterol were significantly improved (Table 3). HbA1c and total cholesterol/HDL-C ratio also improved at 6 months follow up, albeit not to a statistically significant degree. Waist/hip ratio, systolic blood pressure, diastolic blood pressure, total cholesterol, LDL-C, and triglycerides remained unchanged at 6 months.

**Table 3:** Changes in outcome measures from baseline to follow up at 6 months

	N	Mean (SD)		Mean change (SD)	*P*-value
		Baseline	Follow up		
HbA1c (mmol/mol)	73	58.8 (17.9)	57.2 (18.1)	− 1.6 (10.49)	0.196
Weight (kg)	73	80.70 (15.4)	79.40 (15.5)	− 1.28 (3.48)	0.002
BMI (kg/m^2^)	73	31.8 (5.85)	31.2 (5.82)	− 0.52 (1.35)	0.002
Waist circumference (cm)	68	103 (11.85)	101 (12.9)	− 1.95 (4.26)	< 0.001
Hip circumference (cm)	72	107 (11.9)	106 (11.2)	− 1.18 (4.75)	0.039
Waist/hip circumference ratio	72	0.97 (0.08)	0.96 (0.09)	− 0.01 (0.05)	0.191
Systolic BP (mm Hg)	71	137 (15.6)	135 (12.63)	− 1.43 (13.39)	0.371
Diastolic BP (mm Hg)	71	81.7 (9.04)	82.2 (9.67)	0.51 (7.5)	0.567
Total cholesterol (mmol/L)	72	4.34 (1.12)	4.39 (1.05)	0.05 (0.82)	0.573
HDL-C (mmol/L)	71	1.22 (0.29)	1.27 (0.29)	0.06 (0.19)	0.016
LDL-C (mmol/L)	58	2.02 (0.91)	2.09 (0.81)	0.06 (0.69)	0.478
Total cholesterol/HDL-C ratio	71	3.68 (0.97)	3.60 (1.01)	− 0.08 (0.6)	0.289
Triglycerides (mmol/L)	72	2.54 (1.24)	2.68 (1.51)	0.14 (1.49)	0.411
Body fat percentage (%)	71	39.1 (7.88)	38.5 (7.75)	− 0.56 (1.76)	0.009
Self-reported general health	71	3.49 (0.91)	3.21 (0.97)	− 0.28 (1.05)	0.026
WHO-5 score	71	46.6 (23.3)	49.1 (26.4)	2.50 (24.5)	0.397
PAID-5 score	60	9.05 (5.62)	6.47 (5.75)	− 2.58 (5.61)	< 0.001
Fatigue Assessment Scale score	58	31.6 (9.40)	30.6 (10.48)	− 1.02 (7.27)	0.288
SDSCA, median (IQR)					
Healthy diet	72	4 (3–7)	6 (4–7)		0.007
Physical activity	72	4.5 (1–7)	7 (3–7)		0.032
Foot care	72	7 (0–7)	7 (2.5–7)		0.083
Checking blood sugar	71	1 (0–7)	1 (0–3)		0.804
Taking diabetes medication	72	7 (7–7)	7 (7–7)		0.930

*HbA1c* glycated haemoglobin, *BMI* body mass index, *BP* blood pressure, *TC* total cholesterol, *HDL-C* high-density lipoprotein cholesterol, *LDL-C* low-density lipoprotein cholesterol, *BFP* body fat percentage, *WHO-5* World Health Organization-5 Well-Being Index, *PAID* Problem Areas in Diabetes, *SDSCA* Summary of Diabetes Self-Care Activities

Healthy diet and physical activity were significantly improved at 6 months (Table 3). No changes were observed in well-being, fatigue and the self-management activities of foot care and taking diabetes medication at 6 months.

### Attendance

There was a general tendency for frequency of DSMES attendance to be associated with improved outcomes at both the end of the intervention and follow up (Table 4). Some variables reached statistical significance.

**Table 4:** Mean difference in outcomes by participant session attendance

	At intervention end	*P*-value	At 6 months	*P*-value
HbA1c (mmol/mol)	− 0.32	0.164	− 0.92	0.186
Weight (kg)	− 0.16	0.061	− 0.36	0.127
BMI (kg/m^2^)	− 0.07	0.047	− 0.15	0.111
Waist circumference (cm)	− 0.62	0.002	− 0.50	0.082
Hip circumference (cm)	− 0.25	0.177	− 0.06	0.841
Waist/hip circumference ratio	− 0.004	0.026	− 0.005	0.139
Systolic BP (mm Hg)	− 0.33	0.620	0.13	0.856
Diastolic BP (mm Hg)	0.09	0.831	0.07	0.889
Total cholesterol (mmol/L)	− 0.04	0.362	0.001	0.978
HDL-C (mmol/L)	0.001	0.937	0.009	0.462
LDL-C (mmol/L)	− 0.05	0.139	0.005	0.906
Total cholesterol/HDL-C ratio	− 0.02	0.491	− 0.02	0.587
Triglycerides (mmol/L)	− 0.02	0.768	0.03	0.760
Body fat percentage (%)	− 0.06	0.447	− 0.28	0.016
Self-reported general health	− 0.01	0.792	− 0.02	0.714
WHO-5 score	1.85	0.108	0.23	0.887
PAID-5 score	0.31	0.312	− 0.17	0.684
Fatigue Assessment Scale score	0.36	0.372	− 0.44	0.445
SDSCA, median (IQR)				
Healthy diet	− 0.08	0.547	0.01	0.928
Physical activity	0.24	0.074	0.16	0.343
Foot care	0.33	0.027	− 0.06	0.773
Checking blood sugar	− 0.06	0.628	0.23	0.095
Diabetes medication	0.16	0.022	− 0.18	0.077

*HbA1c* glycated haemoglobin, *BMI* body mass index, *BP* blood pressure, *HDL-C* high-density lipoprotein cholesterol, *LDL-C* low-density lipoprotein cholesterol, *WHO-5* World Health Organisation-5 Well-Being Index, *PAID* Problem Areas in Diabetes, *SDSCA* Summary of Diabetes Self-Care Activities

## Discussion

A co-created and culturally sensitive DSMES intervention improved clinical outcomes and self-reported mental and physical health among Urdu-, Turkish- and Arabic-speaking immigrants with type 2 diabetes in Denmark. Several outcomes, including HbA1c, weight, BMI, self-rated general health and the self-management activities of healthy diet and physical activity, significantly improved from pre- to post-intervention and from pre-intervention to follow up at 6 months. In addition, outcomes such as waist and hip circumference and diabetes-related emotional distress that were not significantly different at the end of the intervention were significantly improved at 6 months. In addition, more frequent DSMES session attendance tended to be associated with improved outcomes. Finally, we found very poor psychosocial health among the population at baseline, underscoring the need to consider immigrants from the target group as a hardly reached group that needs DSMES interventions targeted to their needs, perspectives, and preferences.

DSMES interventions targeting immigrants have shown limited effects in reducing HbA1c levels [36]. This could be related to a limited focus on cultural sensitivity in these interventions compared with the present study. A 2014 review showed that culturally appropriate health education targeting ethnic minority groups with type 2 diabetes has short‐ to medium‐term effects on glycaemic control, diabetes knowledge and healthy lifestyles [19]. However, previous studies did not find significant psychosocial improvements such as quality-of-life measures to the same extent we did. Notably, a vast majority (24/33 or 72%) of the review studies were conducted in the US and may not correspond to European and Danish healthcare structures or migration history. Moreover, previous studies also differ from ours in terms of defining and ensuring cultural sensitivity. Most studies do not describe how they provide or ensure cultural sensitivity [36] and the categorisation of cultural appropriateness or culturally sensitivity differs substantially within studies. For example, a review defined cultural appropriateness very broadly with one criterion being the delivery of an intervention to same-sex groups [19]. Notably, a recent UK study of a culturally tailored DSMES intervention drawing on co-design methods and targeting an ethnic minority group showed positive effects on several outcomes, including HbA1c, BMI and physical activity, compared to a control group [37].

### How do we Maintain Effects After 6 Months?

The challenge with lifestyle changes is often maintain them over the long term. Previous studies have found that long-term lifestyle improvements among people with T2D, particularly behavioural changes such as diet and exercise, are often harder to achieve [38, 39]. In our study, many outcomes remained improved at 6 months, and some positive outcomes were only observed at 6 months.

CUSTOM is a complex interdisciplinary intervention consisting of several interacting components and based on person-centred theories, such as the flourishing approach. These characteristics may have contributed to the long-term effects we observed. A recent review similarly found that diabetes self-management educational interventions based on person-centred values, empowerment approach and relevant self-efficacy theory were more successful in obtaining positive outcomes [38]. Another review emphasised the advantage of community-based health promotion, establishing close ties between health care providers and community members [40].

In addition, several educational strategies may explain the positive outcomes we observed, such as using a visual representation of sessions to enhance participant retention; 71% of participants completed at least four sessions. Additional facilitators may have included clarifying the roles of interpreters, educators and participants and articulating why active involvement of the target group was important to benefitting from the intervention. The intervention focused both on physical and psychological aspects of diabetes, recognising the importance of mental health issues. In addition, active involvement of the target group through co-creation during design was likely to have ensured insight into and incorporated preferences of the target population for surface structures and deeper structures and forces influencing health behaviour.

### Strengths and Limitations

Study strengths include a high participation rate, limited loss to follow-up, relatively long follow-up time and the combination of clinical and self-reported outcome measures. It should, however, be noted that HbA1c as a clinical measure of glycaemic control is associated with some uncertainty due to its variability over time and these variations in themselves have a health effect [41, 42]. Another study limitation was the lack of a control group, which precludes exploring causal relations. Performance bias cannot be ruled out because it was impossible to blind participants. The generalisability of our findings may be affected by heterogeneity between and within immigrant groups.

## Conclusion

The co-created and culturally sensitive CUSTOM DSMES program was highly effective in improving short- and longer-term physical and mental health among persons speaking Urdu, Turkish and Arabic with T2D. Co-creation processes ensured the activation of local resources, which is recommended as a means to reducing ethnic health inequities. The theory, methods and tools of the CUSTOM program incorporate the preferences, needs and values of the target groups that would not have been identified or addressed by an exclusively researcher-driven process.

## Data Availability

Data supporting the findings of this study are not publicly available due to privacy concerns of the participants. For further information, contact the corresponding author.

## Code Availability

Code for data cleaning and analysis is available from the corresponding author upon request.
